# Optimal Control Strategy for Abnormal Innate Immune Response

**DOI:** 10.1155/2015/386235

**Published:** 2015-04-08

**Authors:** Jinying Tan, Xiufen Zou

**Affiliations:** ^1^School of Mathematics and Statistics, Wuhan University, Wuhan 430072, China; ^2^College of Science, Huazhong Agricultural University, Wuhan 430070, China

## Abstract

Innate immune response plays an important role in control and clearance of pathogens following viral infection. However, in the majority of virus-infected individuals, the response is insufficient because viruses are known to use different evasion strategies to escape immune response. In this study, we use optimal control theory to investigate how to control the innate immune response. We present an optimal control model based on an ordinary-differential-equation system from a previous study, which investigated the dynamics and regulation of virus-triggered innate immune signaling pathways, and we prove the existence of a solution to the optimal control problem involving antiviral treatment or/and interferon therapy. We conduct numerical experiments to investigate the treatment effects of different control strategies through varying the cost function and control efficiency. The results show that a separate treatment, that is, only inhibiting viral replication (*u*_1_(*t*)) or enhancing interferon activity (*u*_2_(*t*)), has more advantages for controlling viral infection than a mixed treatment, that is, controlling both (*u*_1_(*t*)) and (*u*_2_(*t*)) simultaneously, including the smallest cost and operability. These findings would provide new insight for developing effective strategies for treatment of viral infectious diseases.

## 1. Introduction

The innate immunity plays a critical role in the control of viral infection because it is known that most infectious pathogens are eliminated through the innate immune response without necessarily requiring the activation of adaptive immunity [1–3]. However, virus-mediated innate immune response is a complex dynamical process because multiple signaling pathways are activated in response to virus infection. Interferons (IFNs) are cytokines that are crucial for limiting viral replication at the site of infection and for coordinating adaptive response that leads to the development of antigen-specific acquired immunity. One of the mechanisms by which the innate immune system senses the invasion of pathogenic microorganisms is through the Toll-like receptors (TLRs) [4]. TLR signaling pathways induce the expression of type I IFN in a myeloid differentiation factor 88- (MyD88-) dependent manner, thus promoting the production of many antiviral proteins (such as Mx, ISG20, OAS, and PKR) and immunoactive cytokines. These antiviral components inhibit viral replication and cause apoptosis of infected cells, subsequently resulting in the clearance of the infectious pathogens.

To better understand the dynamics of the innate immune response and the regulation of the signaling components, mathematical modeling and theoretical analyses are increasingly being used to investigate the mechanism of virus-induced signaling pathways [5–8]. To the best of our knowledge, the study about the control of the complex process of virus-mediated innate immune response has not been reported in the literature. Although there have been a few reports of optimal theory to the treatment of the spread of infectious diseases from macroscopic levels [9–13], such as in cell levels [14, 15] or population levels [11], none of these studies has included IFNs and antiviral proteins (AVPs) in virus-mediated signaling pathways as the specific components.

**Figure 1:**
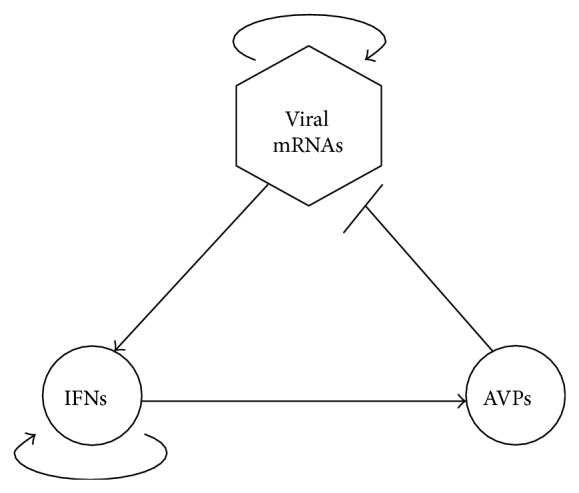
A schematic diagram of interaction among viral mRNAs, IFNs, and AVPs adapted from [16]. A line with an arrow represents positive regulation and a line with a short bar indicates negative regulation.

In a previous study [16], we developed a simplified model of virus-activated signaling pathways to investigate the dynamics and regulation of the innate immune response. In our model, the interactions among the following components are considered, that is, viral mRNAs produced from viral infection, type I IFNs, and AVPs (Figure 1). In the present study, we establish an optimal control model of virus-triggered signaling network and investigate the optimal control strategies for the innate immune response.

This paper is organized as follows.  Section 2 proposes an optimal control model based on an ordinary-differential-equation system from our previous work which was validated by biological experiments [16]. Moreover, the existence of an optimal solution is proven. The results of simulations under different cases and sensitivity analysis are presented in Section 3. Finally, Section 4 summarizes with conclusions.

## 2. Models and Methods

### 2.1. Optimal Control Model

From Figure 1, viruses induce the expression of IFNs, IFNs activate the expression of AVPs, and AVPs inhibit the replication of viral mRNAs, forming a negative feedback loop. Viruses have an autofeedback (or self-replication), and there exists a positive feedback by multistep reactions from IFNs to IFNs. Based on the law of mass action, the production rates (*k*_1_, *k*_2_, and *k*_3_), and degradation rates (*d*_1_, *d*_2_, and *d*_3_) of viral mRNAs, IFNs and AVPs are assumed to be linearly proportional to their concentrations, respectively. The processes of AVPs inhibiting viral mRNAs and the positive feedback of IFNs are represented using Hill functions. Then, we can establish a nonlinear ordinary-differential-equation system (1) with three states (viral mRNAs, IFNs, and AVPs), revised from the virus-triggered innate immune regulatory network studied in [16] by neglecting the time delays in order to simplify the following discussion:

(1) dX(t)dt=k1X(t)·b1K1n1K1n1+Zn1(t)−d1X(t),dY(t)dt=k2X(t)+b2Yn2(t)K2n2+Yn2(t)−d2Y(t),dZ(t)dt=k3Yt−d3Zt,

where *X*(*t*), *Y*(*t*), and *Z*(*t*) are the concentrations of three components, viral mRNAs, IFNs, and AVPs, respectively. *K*_1_ and *K*_2_ are the inhibition and activation coefficients of AVPs and IFNs, respectively; *b*_1_ and *b*_2_ are the maximal production rates; and *n*_1_ and *n*_2_ are the Hill coefficients in two Hill functions, respectively.

To make the theoretical analysis convenient, we nondimensionalize system (1). Time is scaled relative to the degradation rate (*d*_3_) of AVPs. We make the following substitutions and assume that all of the model parameters are greater than 0 for studying the actual biological significance: *x*(*t*) = (*k*_2_ *k*_3_/*K*_1_ *d*_3_^2^)*X*(*t*), *y*(*t*) = (*k*_3_/*K*_1_ *d*_3_)*Y*(*t*), *z*(*t*) = (1/*K*_1_)*Z*(*t*), $\bar{t}=d_{3}t$, *K* = (*k*_3_/*K*_1_ *d*_3_)*K*_2_, *α*_1_ = *k*_1_ *b*_1_/*d*_3_, *α*_2_ = *d*_1_/*d*_3_, *α*_3_ = *k*_3_ *b*_2_/*K*_1_ *d*_3_^2^, *α*_4_ = *d*_2_/*d*_3_, *σ*_1_ = *α*_1_/*α*_2_ = *k*_1_ *b*_1_/*d*_1_, *σ*_2_ = *α*_3_/*α*_4_.

Using *t* instead of $\bar{t}$ for notational convenience, we obtain the nondimensional system of equations:

(2) dxdt=σ1α2x(t)1+zn1(t)−α2x(t),dydt=x(t)+σ2α4yn2(t)Kn2+yn2(t)−α4y(t),dzdt=yt−zt,

where *x*(*t*), *y*(*t*), and *z*(*t*) are the dimensionless concentrations of viral mRNAs, IFNs, and AVPs, respectively. *α*_2_ and *α*_4_ are called the relative degradation rates of the viruses and IFNs with respect to the degradation rate (*d*_3_) of AVPs, respectively. *K* is the relative activation coefficient of IFNs with respect to AVPs. *σ*_1_ can be viewed as the relative ratio between viral replication and degradation, which represents the self-replicating ability of viruses. Similarly, *σ*_2_, the ratio between IFN activation and degradation, can be viewed as the relative strength of IFN production. For brevity and clarity in the study, we let *n*_1_ = *n*_2_ = 2, which leads to system (3) which presents a complex dynamic behavior and is more consistent with the biological experiments [16].

Now, optimal control theory is used to investigate intervention strategy for abnormal innate immune response. Biological evidences have shown that one class of antiviral drugs plays roles through inhibiting and controlling viral replication [17] and another class of drugs are interferons, which stimulate the immune system to attack a range of pathogens [18]. Therefore, through taking into account retarding viral replication or (and) enhancing interferon activity, we propose the following control system:

(3) dxdt=[1−ε1u1(t)]σ1α2x(t)1+z2(t)−α2x(t),dydt=xt+σ2α4y2tK2+y2t−[1−ε2u2(t)]α4y(t),dzdt=yt−zt,

where control function *u*_1_(*t*) represents external forces (e.g., inhibiting viral replication by drug treatment) to reduce viral replication ratio *σ*_1_, which is weakened to [1 − *ε*_1_ *u*_1_(*t*)]*σ*_1_. Control function *u*_2_(*t*) is desirable to lower the degradation rate of IFNs (e.g., improving interferon activity by interferon therapy), which will in turn enhance the relative strength of self-replication of IFNs *σ*_2_, making it to be (1/(1 − *ε*_2_ *u*_2_(*t*)))*σ*_2_, where *u*_*i*_(*t*)  (*i* = 1,2) satisfies 0 ≤ *u*_*i*_(*t*) ≤ 1. *u*_*i*_(*t*) = 0 indicates that we do not take any control measures and the host restores health from infection status by his own immunomodulation. When *u*_*i*_(*t*) = 1, we need to do our best to interfere with viral infections [13]. Factor *ε*_*i*_  (*i* = 1,2) denotes the control implementation efficiency, meeting 0 < *ε*_*i*_ < 1. For the convenience of discussion, we assume that *ε*_1_ = *ε*_2_ [11].

### 2.2. Performance Function and Control Strategies

Obviously, system (3) presents a complex coupling system, so performance function (objective function) *J* is determined by minimizing the treatment cost and the number of virus particles in a finite time interval [0, *t*_*f*_] based on optimal control theory

(4) $$\begin{array}{c} J\left(u_{1}\left(t\right),u_{2}\left(t\right)\right)=\overset{t_{f}}{\underset{0}{\int }}\left(Cx+\frac{W_{1}}{2}u_{1}^{2}\left(t\right)+\frac{W_{2}}{2}u_{2}^{2}\left(t\right)\right)dt. \end{array}$$

As it is customary, we choose to model the control efforts with a combination of quadratic terms, *u*_*i*_^2^(*t*) (*i* = 1,2). The constants *C* and *W*_*i*_ (*i* = 1,2) are weights of virus state and control function *u*_*i*_(*t*) (*i* = 1,2) over [0, *t*_*f*_], respectively. In order to simplify the following study, we suppose that control *u*_1_(*t*) has the same importance as *u*_2_(*t*); that is, *W*_1_ = *W*_2_. The optimal control problem is to find optimal functions (*u*_1_^∗^(*t*), *u*_2_^∗^(*t*)) such that

(5) $$\begin{array}{c} J\left(u_{1}^{*}\left(t\right),u_{2}^{*}\left(t\right)\right)=\underset{\Omega }{\min}J\left(u_{1}\left(t\right),u_{2}\left(t\right)\right), \end{array}$$

where *Ω* = {*u*_1_(*t*), *u*_2_(*t*)∈(*L* ^2^(0, *t*_*f*_))^2^∣0 ≤ *u*_1_(*t*), *u*_2_(*t*) ≤ 1, *t* ∈ [0, *t*_*f*_]} subject to the state equations (2) and appropriate initial conditions.

Three different control strategies are investigated. This approach can be used to test various options. Here, we only look at how the state variables change under the following three alternatives.

- Strategy 1: inhibit viral replication by reducing (blocking) virus production (control *u*_1_(*t*) alone).
- Strategy 2: enhance the activity of IFN to inhibit virus by reducing the relative degradation rate of IFN (control *u*_2_(*t*) alone).
- Strategy 3: two treatment controls on clinically infectious cases (controls *u*_1_(*t*) and *u*_2_(*t*)).

### 2.3. Theoretical Analysis

First, system (3) is transformed into the problem of minimizing the following Hamiltonian function of optimal control problem:

(6) H=Cx+W12u12(t)+W22u22(t)+λ1t1−ε1u1σ1α2xt1+z2t−α2xt+λ2txt+σ2α4y2tK2+y2t−1−ε2u2α4yt+λ3tyt−zt.

From Pontryagin's Maximum Principle [19], we obtain the following theorem for assuring the existence of a solution to our optimal control problem.

**Theorem 1 .**

If the continuous functions *λ*_*i*_(*t*), (*i* = 1,2, 3) satisfy adjoint equations

(7) dλ1dt=−C−λ1t1−ε1u1tσ1α21+z2t−α2−λ2(t),dλ2dt=−λ2t2σ2α4K2ytK2+y2t2−1−ε2u2tα4 −λ3(t),dλ3dt=−λ1(t)2[1−ε1u1(t)]σ1α2x(t)z(t)1+z2t2+λ3(t)

and transversality conditions (terminal conditions)

(8) $$\begin{array}{c} \lambda_{i}\left(t_{f}\right)=0,\;i=1,2,3. \end{array}$$

Then, there exist optimal controls (*u*_1_^∗^(*t*), *u*_2_^∗^(*t*)) and corresponding solutions (*x* ^∗^(*t*), *y* ^∗^(*t*), *z* ^∗^(*t*)) that minimize *J*(*u*_1_(*t*), *u*_2_(*t*)) over *Ω*. Moreover,

(9) u1∗(t)=min⁡max⁡0,ε1σ1α2λ1xW11+z2,1,u2∗(t)=min⁡max⁡0,−ε2α4λ2yW2,1.

**Proof**

From Corollary 4.1 of [20] and Theorem 1 of [11], there are optimal controls because the integrand of *J* is a convex function of (*u*_1_(*t*), *u*_2_(*t*)). Also, system (3), also called state equations, satisfies the Lipshitz property with respect to the state variables because state solutions are *L* ^*∞*^ bounded. The following results can be derived from Pontryagin's Maximum Principle [19]:

(10) $$\begin{array}{c} \frac{d\lambda_{1}\left(t\right)}{dt}=-\frac{\partial H}{\partial x},\\ \frac{d\lambda_{2}\left(t\right)}{dt}=-\frac{\partial H}{\partial y},\\ \frac{d\lambda_{3}\left(t\right)}{dt}=-\frac{\partial H}{\partial z}. \end{array}$$

So we obtain system (7), and at the optimal controls and corresponding states, we have

(11) $$\begin{array}{c} \lambda_{i}\left(t_{f}\right)=0,\;i=1,2,3. \end{array}$$

The differentiations of *H* with respect to *u*_1_ and *u*_2_ at optimal controls are all 0:

(12) dHdu1u1=u1∗=W1u1∗−λ1ε1σ1α2x1+z2=0,dHdu2u2=u2∗=W2u2∗+λ2ε2α4y=0.

Then, we obtain *u*_1_^∗^(*t*) = *ε*_1_ *σ*_1_ *α*_2_ *λ*_1_ *x*/*W*_1_(1 + *z* ^2^) and *u*_2_^∗^(*t*) = −*ε*_2_ *α*_4_ *λ*_2_ *y*/*W*_2_. By taking into account the control bounds, the properties (9) hold up.

### 2.4. Numerical Simulations

Based on optimal control theory, the solution of the optimal control problem (5) can be transformed into solving the following two-point boundary value problem:

(13) dxdt=[1−ε1u1(t)]σ1α2x(t)1+z2(t)−α2x(t),dydt=x(t)+σ2α4y2(t)K2+y2(t)−[1−ε2u2(t)]α4y(t),dzdt=y(t)−z(t),dλ1dt=−C−λ1t1−ε1u1tσ1α21+z2t−α2 −λ2(t),dλ2dt=−λ2t2σ2α4K2ytK2+y2t2−1−ε2u2tα4 −λ3(t),dλ3dt=−λ1(t)2[1−ε1u1(t)]σ1α2x(t)z(t)1+z2t2+λ3(t),u1∗(t)=min⁡{max⁡{0,ε1σ1α2λ1xW1(1+z2)},1},u2∗(t)=min⁡{max⁡{0,−ε2α4λ2yW2},1},s(0)=(x(0),y(0),z(0))=(x0,y0,z0),λ(tf)=(λ1(tf),λ2(tf),λ3(tf))=(0,0,0).

Then, we carry out numerical simulations for system (13) under three different strategies described in Section 2.2. We solve by iterating the nonlinear optimal control system consisting of state equation (3), that is, the first three equations of system (13), and adjoint equation (7), from the fourth to sixth equation of system (13). State equation (3) is numerically computed forward in a given initial conditions, and the corresponding adjoint equation (7) is solved backward in given transversality condition (8). Iterating process is not terminated until it meets the convergence criteria specified in advance (10^−5^). Here, we can iteratively solve these problems by the steepest descent method [21, 22].

**Step 1 .**

Initialize *t*_*f*_, *N*, *s*(0), *λ*(*t*_*f*_), and *i* = 0, subdivide [0, *t*_*f*_] into *N* equal subintervals, and set tolerance *γ*, step size *τ*, and *u* ^(0)^(*t*) = *u* ^(0)^(*t*_*k*_) = 1, *t* ∈ [*t*_*k*_, *t*_*k*+1_], *k* = 0,1,…, *N* − 1.

**Step 2 .**

For *i*, integrate state equations (3) from 0 to *t*_*f*_ with *s*(0) and *u* ^(*i*)^(*t*) and save the state trajectory *s* ^(*i*)^(*t*) and integrate adjoint equations (7) backward, that is, from *t*_*f*_ to 0, with *λ*(*t*_*f*_).

**Step 3 .**

Calculate

(14) $$\begin{array}{c} {\left\|\frac{\partial H^{\left(i\right)}}{\partial u}\right\|}^{2}≐\overset{t_{f}}{\underset{0}{\int }}{\left\|\frac{\partial H^{\left(i\right)}}{\partial u}\right\|}^{T}\left\|\frac{\partial H^{\left(i\right)}}{\partial u}\right\|dt. \end{array}$$

If ‖∂*H* ^(*i*)^/∂*u*‖ ≤ *γ*, then stop the iterative procedure, and output the extremal state and control. Otherwise, renew the control function by *u* ^(*i* + 1)^(*t*_*k*_) = *u* ^(*i*)^(*t*_*k*_) − *τ*(∂*H* ^(*i*)^/∂*u*)(*t*_*k*_), *k* = 0,1,…, *N* − 1. Replace *u* ^(*i*)^ by *u* ^(*i*+1)^ and go to Step 2.

The initial values of state variables and the model parameter values are shown in Table 1.

## 3. Results and Discussions

### 3.1. Baseline Cases

**Figure 2:**
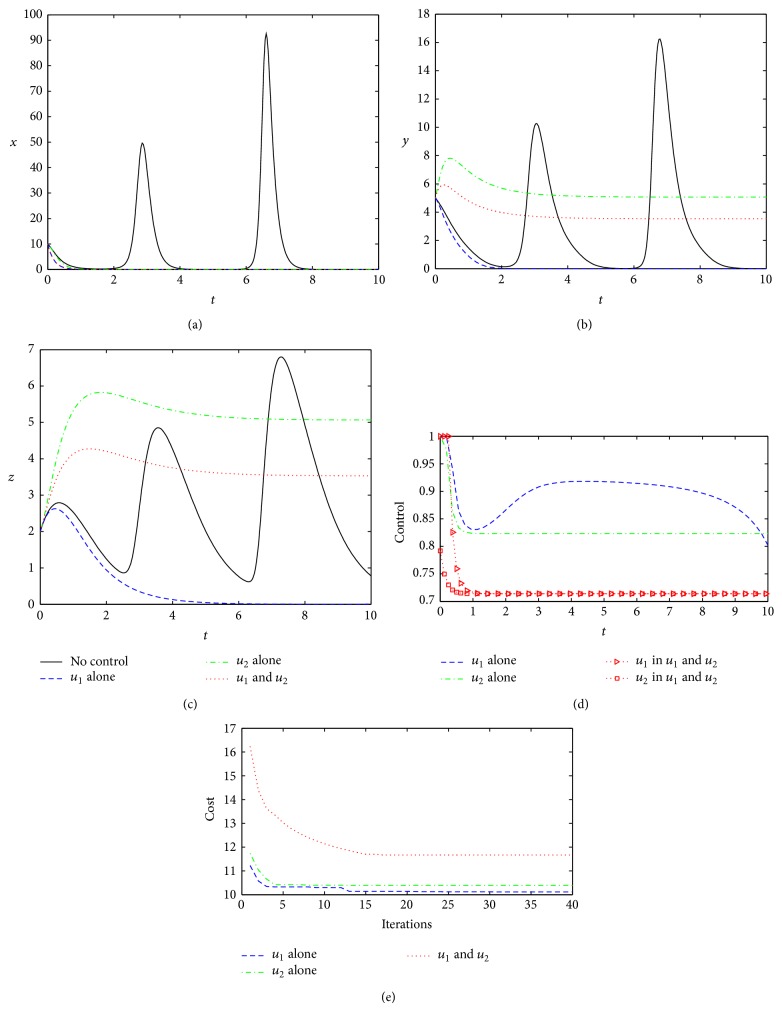
Optimal controls under three different strategies. Black solid lines, in (a), (b), and (c), represent the dimensionless concentration of viral mRNAs (*x*), IFNs (*y*), and AVPs (*z*) without treatment, respectively. Dashed blue line, green dotted line, and red dotted line represent the control effect about *x*, *y*, and *z* under Strategy 1 (only control *u*_1_^∗^), Strategy 2 (only control *u*_2_^∗^), and Strategy 3 (control *u*_1_^∗^ and *u*_2_^∗^), respectively. (d) shows the control functions under the different control methods, in which the red triangle dotted line and squares dotted lines represent control strength of *u*_1_^∗^ and *u*_2_^∗^ when *u*_1_^∗^ and *u*_2_^∗^ are joint action. (e) shows dimensionless costs under the different iterations. All parameters and initial conditions are taken from Table 1.

**Figure 3:**
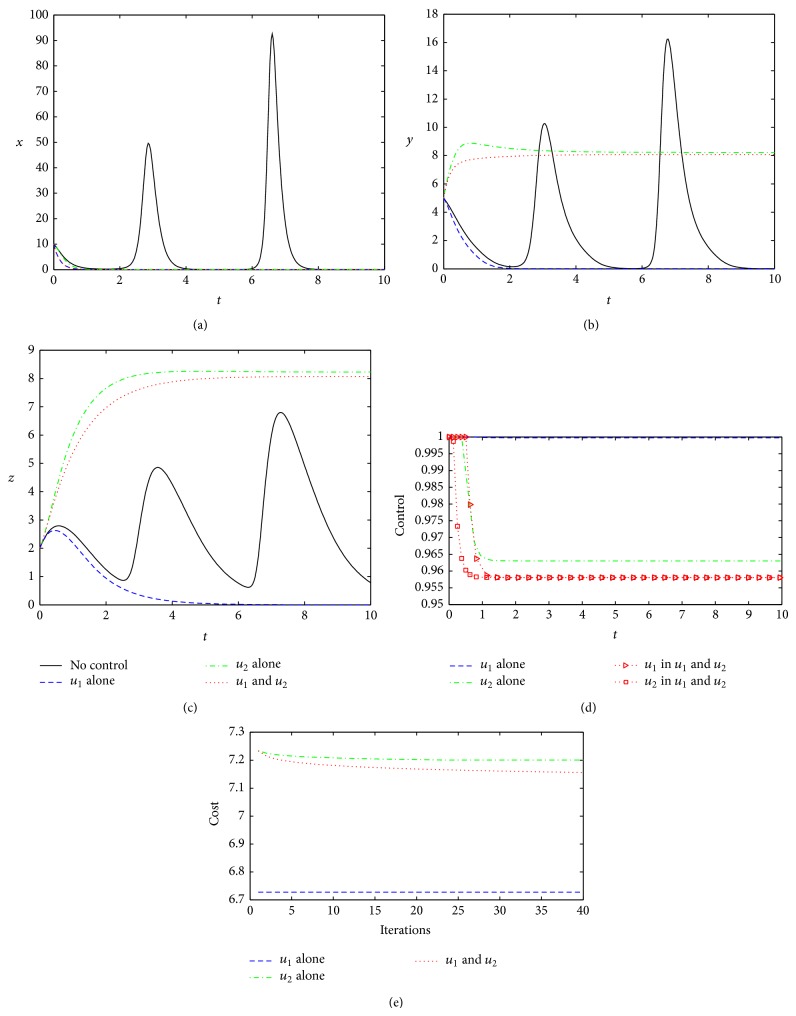
Optimal controls under three different strategies when *W*_1_ = *W*_2_ = 0.1. The legends are set to be the same as in Figure 2. Other parameters and initial conditions are taken from Table 1.

**Figure 4:**
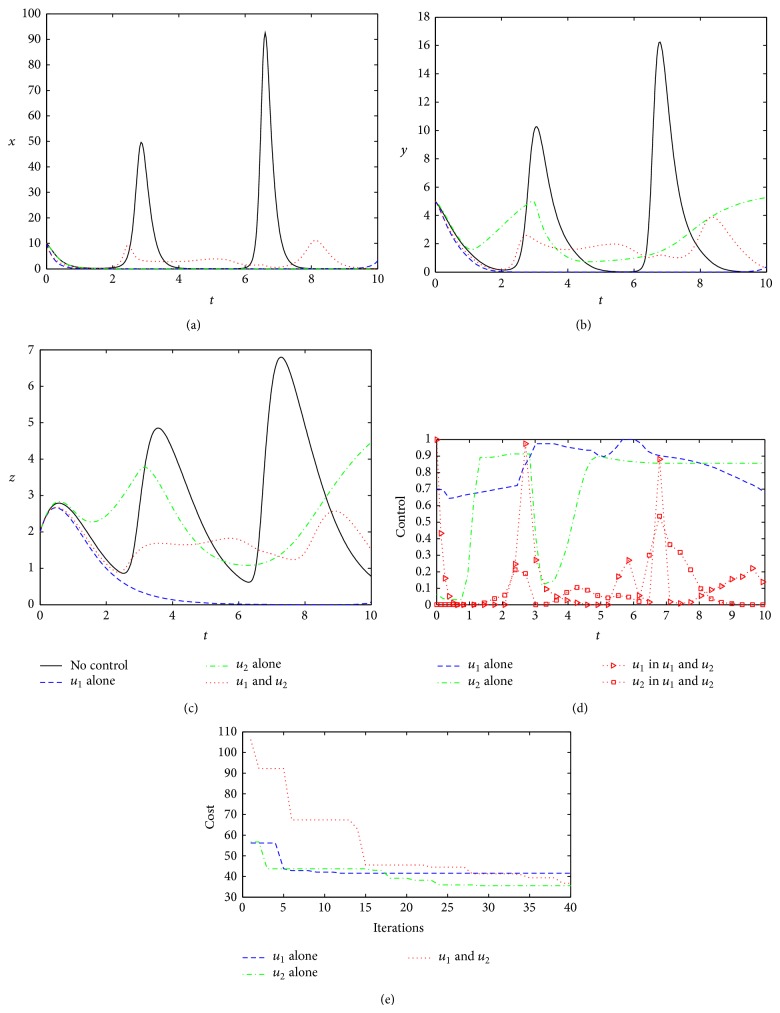
Optimal controls under three different strategies when *W*_1_ = *W*_2_ = 10. The legends are the same as in Figure 2. Other parameters and initial conditions are taken from Table 1.

**Figure 5:**
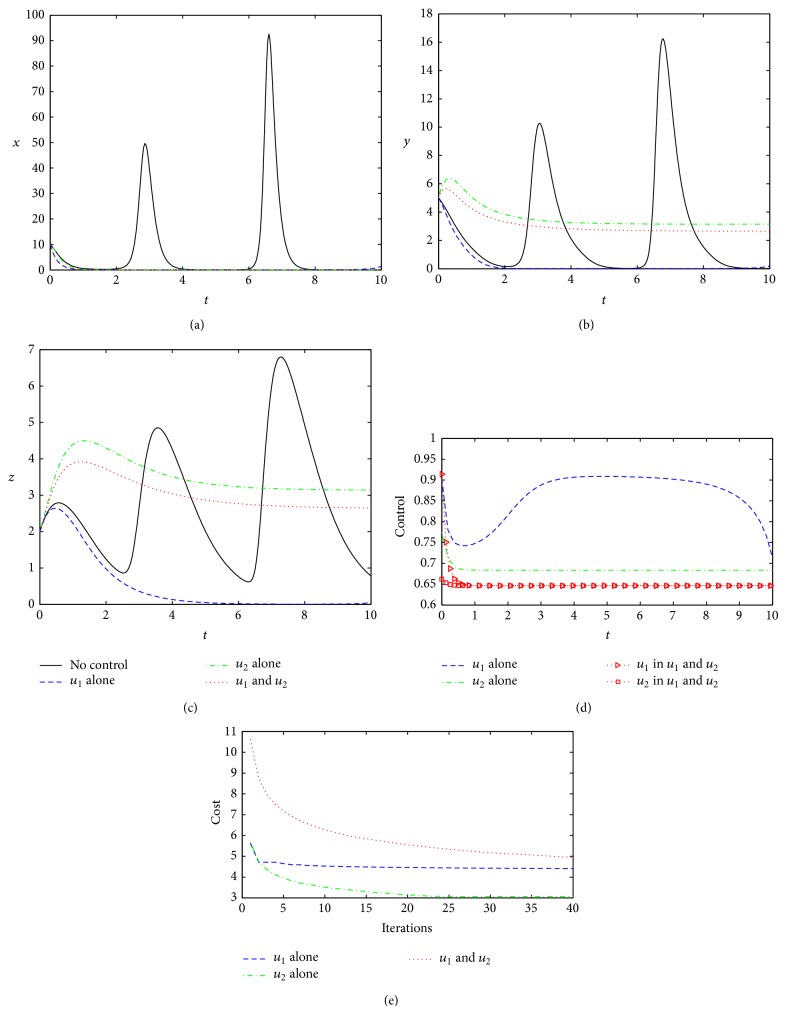
Optimal controls under three different strategies when *C* = 0.1. The legends are the same as in Figure 2. Other parameters and initial conditions are taken from Table 1.

**Figure 6:**
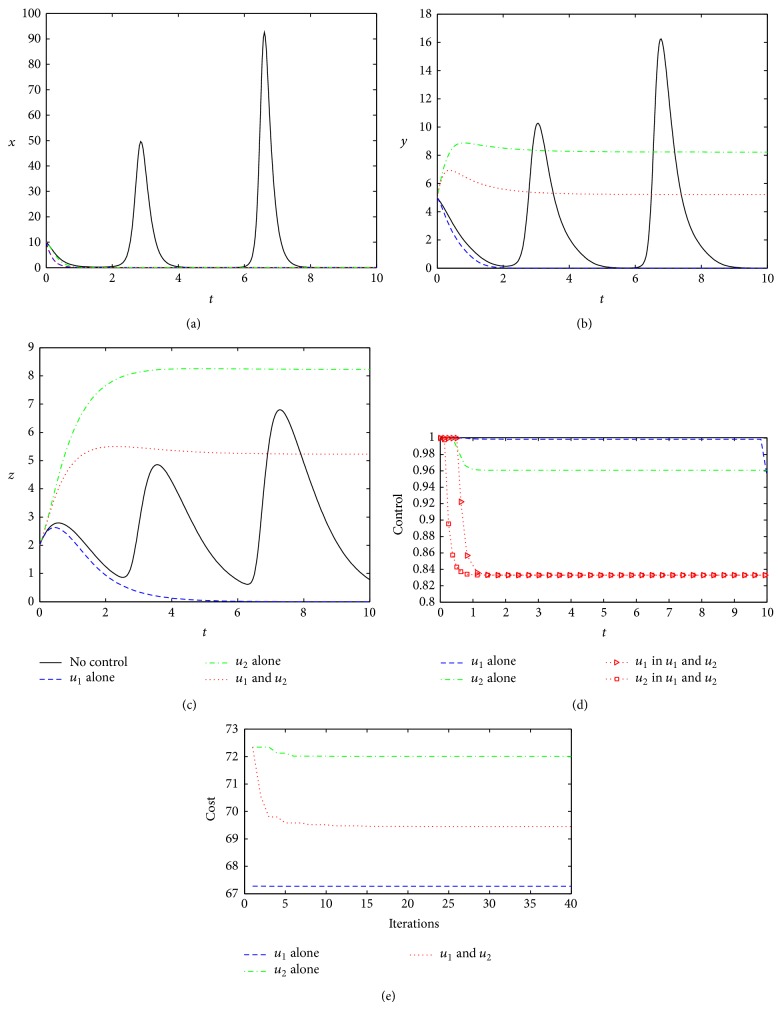
Optimal controls under three different strategies when *C* = 10. The legends are the same as in Figure 2. Other parameters and initial conditions are taken from Table 1.

**Figure 7:**
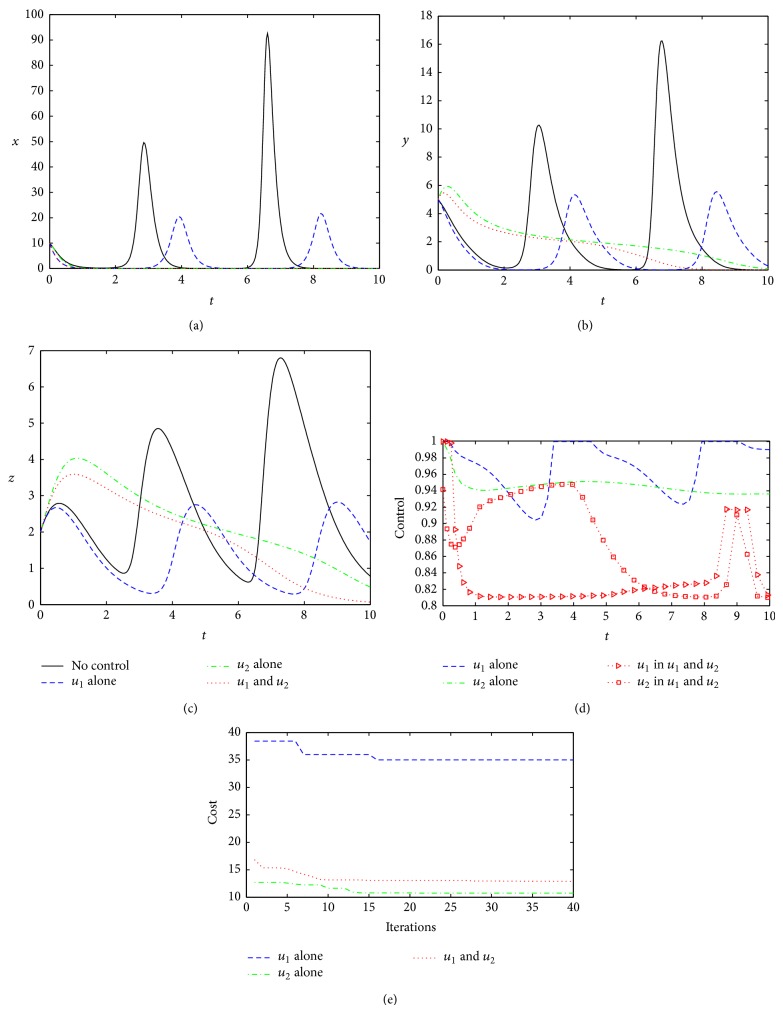
Optimal controls under three different strategies when *ε*_1_ = *ε*_2_ = 0.5. The legends are the same as in Figure 2. Other parameters and initial conditions are taken from Table 1.

**Figure 8:**
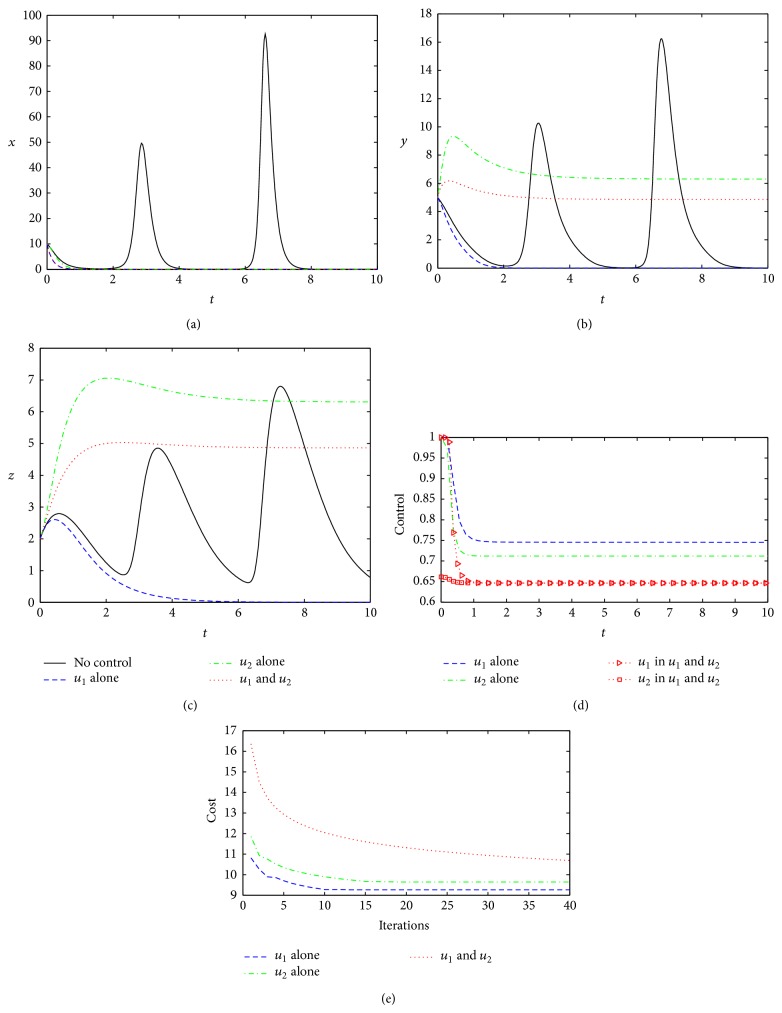
Optimal controls under three different strategies when *ε*_1_ = *ε*_2_ = 1. The legends are the same as in Figure 2. Other parameters and initial conditions are taken from Table 1.

From stability conditions in [16, 23], if no treatment measures are taken, namely, *ε*_1_ = *ε*_2_ = 0, system (3), degenerated into system (2), will produce periodic oscillations under the other model parameters taken from Table 1, which exhibit disease state (solid black lines shown in Figures 2–8—(a), (b), and (c)). A “good” control or treatment is not only to eliminate the virus, but also to make the cost as low as possible. All numerical simulations are implemented using MATLAB 2009b (The MathWorks, Natick, MA).

We now examine the optimal controls under three different control strategies using the model parameters taken from Table 1 unless otherwise stated. The weight coefficients take *C* = 1, and *W*_1_ = *W*_2_ = 1. The treatment efficiency factors *ε*_1_ = *ε*_2_ = 0.8. The length of treatment time is assumed to be 10 time units. Obviously, if no appropriate measures are taken in this course, the viruses will appear in the explosive oscillations and the host will be seriously intrusive or suffer from a disease unavoidably. Under the model parameters from Table 1, Figure 2 shows the three effects of different kinds of controls.

From Figure 2, all three control strategies are able to play virucidal effects (the concentration of viral mRNAs is reduced to 0 quickly), but they also exhibit some differences. For performance function, the optimal cost under Strategy 1 is lower than that produced by Strategy 2 or Strategy 3 (Figure 2(e)), which indicates that optimal control *u*_1_^∗^ under Strategy 1 is more effective than optimal control *u*_2_^∗^ under Strategy 2 or optimal controls *u*_1_^∗^ and *u*_2_^∗^ under Strategy 3 in the entire control process. Through careful analysis, it is not difficult to find that optimal cost is not only related with the control function itself, but also related with the control process (or control effect, e.g., whether *x* rapidly approaches zero?). Also, from simulation results, although the two control functions *u*_1_^∗^ and *u*_2_^∗^ under Strategy 3 are all relatively small (Figure 2(d)), the optimal cost by Strategy 3 is larger than that by Strategy 1 or Strategy 2 (Figure 2(e)). This result indicates that the joint effect of two different controls (Strategy 3) does not necessarily take a more superior to a certain kind of treatment alone. This is an interesting phenomenon, meaning that sometimes the combined effect of multitreatments simultaneously is not necessarily a good idea. Meanwhile, under Strategy 1, interferon and antiviral protein can be rapidly returned to normal levels (*y* = *z* = 0, Figures 2(b) and 2(c)). Therefore, based on the model parameters from Table 1, the control under Strategy 1 is the best choice; that is, the treatment by suppressing viral replication (such as viral suppression by injecting or taking drugs in patients) will receive good therapeutic effect and cost savings.

In addition, it can be seen from Figure 2(e), with the increase of iterations in the algorithm, that the costs will be gradually reduced and be finally stabilized.

### 3.2. The Control Effects When the Cost Function Is Changed

In the actual treatment process restricted by medical conditions, however, the weighting coefficients of performance function will be different. We further examine what different results will show under three different control strategies when weighting coefficients vary.

First, assuming that the weight of viral state is unchanged (*C* = 1), we investigate the effects of control weights *W*_1_ and *W*_2_ on the optimal control.

When the control weighting coefficients become smaller (*W*_1_ = *W*_2_ = 0.1), which indicate that there are less controls, all control manners can also effectively clear the virus (Figure 3(a)), which is the same as in the base case. However, since the control weighting coefficients become smaller, the optimal controls of the three strategies are stronger compared to the base case, and *u*_1_^∗^ is almost 1 under Strategy 1 from Figure 3(d), but the costs have significantly decreased (Figures 3(e) and 2(e)). On the contrary, the cost resulting from optimal control under Strategy 3 is less than that by Strategy 2, indicating that the common effect of combined with *u*_1_^∗^ and *u*_2_^∗^ is better than the use of *u*_2_^∗^ alone. This shows that the reduction in the control weight coefficients, namely, medical conditions likely to be met, taking various measures will be more effective. However, on the overall situation, the optimal control from Strategy 1 is more effective than that from Strategy 2 or Strategy 3 because the cost from Strategy 1 is lowest among three strategies (Figure 3(e)) and the treatment from Strategy 1 is more efficient (*u*_1_^∗^ ≈ 1, Figure 3(d)) than that from Strategy 2 or Strategy 3.

When the control weighting coefficients become larger (*W*_1_ = *W*_2_ = 10), compared with the base case, control effects are different under the three different control strategies. Strategy 1 and Strategy 2 are able to clear the virus completely, but Strategy 3 cannot (Figure 4(a)). Under Strategy 3, the concentration of the virus shows periodic oscillation; therefore, the host still suffers from illness. Although there is a substantial reduction in the optimal cost under Strategy 3, almost to a minimum (comparing with Strategy 2, Figure 4(e)), it can be seen that the optimal control under Strategy 3 is hardly workable because of the optimal controls *u*_1_^∗^ and *u*_2_^∗^ oscillation (Figure 4(d)). Comparing Strategy 1 to Strategy 2, it can found that when the control weights become larger, although the total cost increases in all the two strategies (almost 3-4 times to the base case), the second control shows better performance, resulting in the lowest cost (Figure 4(e)). However, from the operability, the optimal control under Strategy 1 is easier to be implemented than that under Strategy 2 or Strategy 3 because the other two controls are too turbulent (Figure 4(d)), and interferon and antiviral proteins can be returned to normal (*y* = *z* = 0, Figures 4(b) and 4(c)). Therefore, Strategy 1 is more feasible and effective, but to be expected, Strategy 2 will become a more realistic choice when the control weight coefficients are increased.

Secondly, we discuss what the effect on the optimal control will be, assuming that the control weights are unchanged (*W*_1_ = *W*_2_ = 1), when viral status weight becomes smaller (*C* = 0.1) or larger (*C* = 10).

When the virus status weighting coefficient becomes smaller (*C* = 0.1), the same as the basic situation, three control methods are able to effectively clear the virus (Figure 5(a)) and the total costs will effectively reduce (Figure 5(e)), saving even more than that due to the control weight coefficients decreasing. On the one hand, the low costs result from the weight coefficient reduced. On the other hand, the optimal control intensity under three strategies has reduced in different degrees (Figures 2(d) and 5(d)). It can be found that, moreover, Strategy 2 will lead to the lowest cost, almost by half of the other two strategies. So, it can be seen that when the viral weight coefficient decreases constantly, taking measures to enhance the interferon activity will increasingly become a good choice.

When the viral status weight coefficient becomes larger (*C* = 10), the control effect of three control methods is similar to the base case, but the control intensity is much larger (Figure 6(d)) and the total costs are much higher (almost 7-8 times than basic case, Figure 6(e)). Also, Strategy 1 has significant advantages compared to the other two strategies, being the best choice again. Therefore, it can be expected that when viral status weight coefficient becomes larger and larger, the independent treatment of blocking viral replication will be more beneficial to treat viral diseases.

In summary, when the weights are changed, Strategy 3 (cotreatment) would not be a good choice and Strategy 1 or Strategy 2 becomes the alternative. Wherein, when the control weights become smaller or viral weight becomes larger, the best therapy would be the treatment resulted from Strategy 1. By contrast, when the control weights become larger or viral weight becomes smaller, the best therapy would be resulted from Strategy 2. This shows that, in the treatment of disease, the separate treatment may sometimes have an advantage in terms of cost and operability.

### 3.3. The Control Effects When Treatment Efficiency Factors Are Changed

Next, we examine how to change about the control effects under the three different strategies when treatment efficiency factors are varied.

When the treatment efficiency factors become smaller (*ε*_1_ = *ε*_2_ = 0.5), it can be seen that the optimal control under Strategy 1 is to be the worst choice, which not only produces the highest cost and weakens operability because of large fluctuations about *u*_1_^∗^ but also cannot eradicate the virus, showing periodic oscillation. The result causes too serious illness for the host to recover (Figure 7). Although the optimal cost under Strategy 3 is smaller than that of Strategy 1, the optimal control also shows large fluctuation characteristics, meaning not good maneuverability (Figure 7(d)). Contrary to the two kinds of optimal control strategies, the optimal control *u*_2_^∗^ will generate the lowest cost and good operability during the entire treatment process (Figure 7(d)). Therefore, when the treatment efficiency factors become smaller, Strategy 2 by enhancing interferon activity becomes the best treatment option in turn.

When the treatment efficiency factors become larger (*ε*_1_ = *ε*_2_ = 1), compared with the base case, the optimal costs under three different control modes have different degrees of reduction and the control intensity has also been weakened. The optimal control under Strategy 1 again becomes the best choice (Figure 8(e)), which shows that increasing the treatment efficiency factors will be beneficial to Strategy 1 to achieve good results. Moreover, it can be seen that increasing treatment efficiency factors will reduce the volatility of the optimal control and will enhance treatment options operability (Figure 8(d)). Therefore, improving the therapeutic efficiency factors for the actual treatment of disease will not only reduce costs, but also increase the choice of treatment.

### 3.4. Sensitivity Analysis

A multivariate uncertainty and sensitivity analysis are performed to investigate the effects of input parameters (*σ*_1_, *σ*_2_, *α*_2_, *α*_4_, and *K*) on the optimal cost by Monte Carlo method [24, 25]. The five parameters are perturbed by 20% at the values from Table 1, assuming that the input parameters are mutually independent.

**Figure 9:**
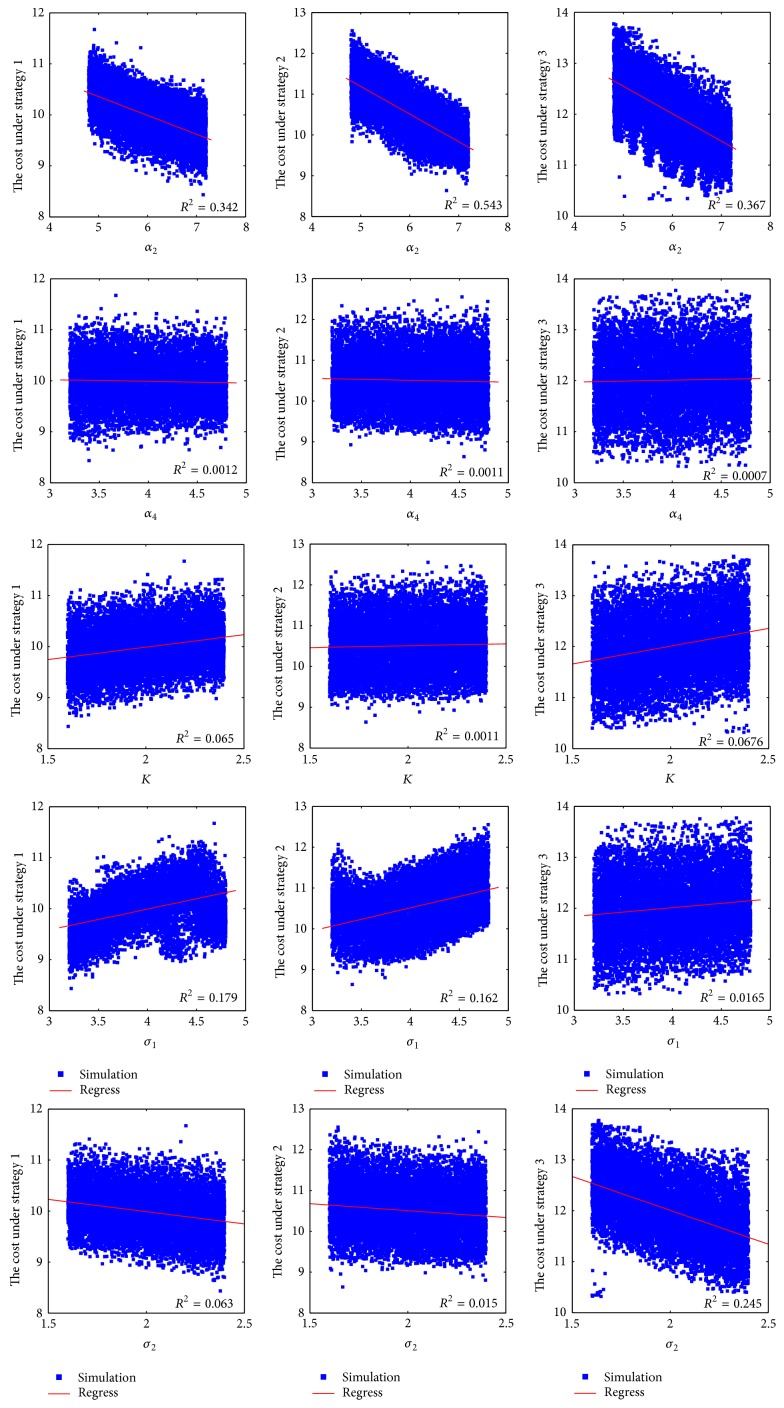
Simulations and Pearson's linear correlation coefficients.

We sampled ranges of the parameters 10000 times using random sampling to yield 10000 scenarios. Then we conducted multivariate uncertainty and sensitivity analysis to determine the uncertainty in the optimal cost that is due to the uncertainty in estimating the input parameters. From Figure 9, we can observe that almost all of Pearson linear correlation coefficients are small except a few coefficients. The reason is because, on the one hand, the linear correlation between the parameters and the optimal cost is weak and the parameters interfere with each other on the other hand.  Table 2 provides Spearman partial rank correlation coefficients (SPRCCs) for the optimal cost.

From sensitivity analysis in Table 2, we found that the SPRCCs are all statistically significant; that is, *P* ≪ 0.05, for all strategies. The most statistically influential inputs in Strategy 1 and Strategy 2 are *α*_2_ and *σ*_1_ (|SPRCC| > 0.5) while the other three parameters are less influential. In the three control strategies, the trend of the impact of parameters on the optimal costs is the same; that is, a decrease in *α*_2_ or *σ*_2_ corresponds to an increase in the optimal costs while an increase in *σ*_1_ or *K* corresponds to an increase in the optimal costs. SPRCC of parameter *α*_4_ in the three strategies is very small, meaning that the impact of *α*_4_ on optimal cost is small.

## 4. Conclusions

We study which optimal control should be used to achieve the best antiviral treatment effect under three different strategies based on optimal control theory and we have the following conclusions (summary in Table 3).

In the basic case (all parameters taken from Table 1), the three control strategies can effectively kill the viruses, but Strategy 1 will be the best treatment option, which not only leads to the smallest cost but also makes the antiviral proteins and interferons quickly return to normal levels.

When the weights vary, Strategy 1 or Strategy 2 will become the best optional control. When the control weights are small or viral status weight becomes large, Strategy 1 will still be the best treatment option. On the contrary, when the control weights increase or viral status weight decreases, the optimal control will be Strategy 2.

When the treatment efficiency factors decrease, the optimal control by enhancing interferon activity is Strategy 2, yielding the best treatment options while minimizing cost and reducing control volatility throughout the treatment period. When treatment efficiency factors increase, Strategy 1 again becomes the best choice.

In all discussed cases, Strategy 3 is unable to become the best treatment, indicating that a separate treatment sometimes has more advantages, including the smallest cost and operability, in the treatment of the disease. Also, improving efficiency factors for treatment will not only reduce the cost, but also provide more the treatment choices in the actual treatment of diseases.

From sensitivity analysis, we found that parameter *α*_2_, the relative degradation rate of virus, is very important for the optimal costs in the three strategies. An increase in *α*_2_ is help to a decrease in the optimal cost.

More elaborate numerical simulations for unequal weight coefficients will be conducted in the further work. In addition, the influences of noises on optimal controls are also investigated in our next research [26]. The above conclusions are based on the theoretical analysis and numerical simulation, the reliability needed to pass inspection by clinical trials, but certainly provide valuable guidance and insight for developing effective treatment of viral infectious diseases.

## Figures and Tables

**Table 1:** The initial values of state variables and the model parameter values.

Parameter	Description	Value
*σ*_1_	Relative ratio between viral replication and degradation	4
*σ*_2_	Relative strength of IFNs production	2
*α*_2_	Relative degradation rates of the viruses	6
*α*_4_	Relative degradation rates of the IFNs	4
*K*	Relative activation coefficient of IFNs	2
*n*_1_	Hill coefficient	2
*n*_2_	Hill coefficient	2
*ε*_1_	Efficiency factor of control *u*_1_	0.8
*ε*_2_	Efficiency factor of control *u*_2_	0.8
*C*	Weight of virus state	1
*W*_1_	Weight of control *u*_1_	1
*W*_2_	Weight of control *u*_2_	1
(*x*(0), *y*(0), *z*(0))	Initial values of state variables	(10,5, 2)

**Table 2:** Spearman partial rank correlation coefficents (SPRCCs).

Parameter		*α*_2_	*α*_4_	*K*	*σ*_1_	*σ*_2_
Strategy 1	SPRCC	−0.6819	−0.0645	0.3559	0.5532	−0.3696
	*P* value	<0.0001	<0.0001	<0.0001	<0.0001	<0.0001
	Rank	1	5	4	2	3

Strategy 2	SPRCC	−0.8100	−0.0639	0.0433	0.5814	−0.2226
	*P* value	<0.0001	<0.0001	<0.0001	<0.0001	<0.0001
	Rank	1	4	5	2	3

Strategy 3	SPRCC	−0.7394	0.0384	0.4054	0.2040	−0.6605
	*P* value	<0.0001	<0.0001	<0.0001	<0.0001	<0.0001
	Rank	1	5	3	4	2

**Table 3:** Optimal strategy when  *C*, *W*_*i*_, or *ε*_*i*_ (*i* = 1,2) change.

	*C*	*W*_1_ = *W*_2_	*ε*_1_ = *ε*_2_	Optimal strategy
Baseline case	1	1	0.8	Strategy 1

*W*_*i*_ change	1	0.1	0.8	Strategy 1
	1	10	0.8	Strategy 2

*C* change	0.1	1	0.8	Strategy 2
	10	1	0.8	Strategy 1

*ε*_*i*_ change	1	1	0.5	Strategy 2
	1	1	1	Strategy 1
